# Can HPV Selfy be considered as a clinically validated HPV test for use in cervical cancer screening?

**DOI:** 10.1186/s12967-022-03627-w

**Published:** 2022-09-16

**Authors:** Marc Arbyn, Jesper Bonde, Kate Cushieri, Mario Poljak

**Affiliations:** 1 Unit of Cancer Epidemiology, Belgian Cancer Centre, Sciensano, J. Wytsmanstreet 14, 1050 Brussels, Belgium; 2 Department of Human Structure and Repair, Faculty of Medicine and Health Sciences, University of Ghent, Ghent, Belgium; 3 Molecular Pathology Laboratory, Department of Pathology, Copenhagen University Hospital, Hvidovre, Denmark; 4 Scottish HPV Reference Laboratory, Royal Infirmary of Edinburgh, Edinburgh, Scotland UK; 5 Institute of Microbiology and Immunology, Faculty of Medicine, University of Ljubljana, Ljubljana, Slovenia

**Keywords:** Human papillomavirus, Cervical cancer screening, Diagnostic test accuracy, Validation of tests

In primary cervical cancer screening, it is crucial to use only hrHPV tests that are clinically validated according to international guidelines in order to reduce the risks of missing relevant disease and of over-treatment. In the recent *J Transl Med *[1] paper, Avian et al. concluded that the *HPV Selfy* assay (Ulisse BioMed, Trieste, Italy) fulfils international validation criteria for hrHPV testing on clinician-collected cervical samples (Meijer guidelines) [2] as well as by extension on self-collected vaginal samples (VALHUDES) [3]. Our perception is that the study by Avian et al. has certain limitations that are worthy of consideration and which may call into question certain conclusions.

**Fig. 1:**
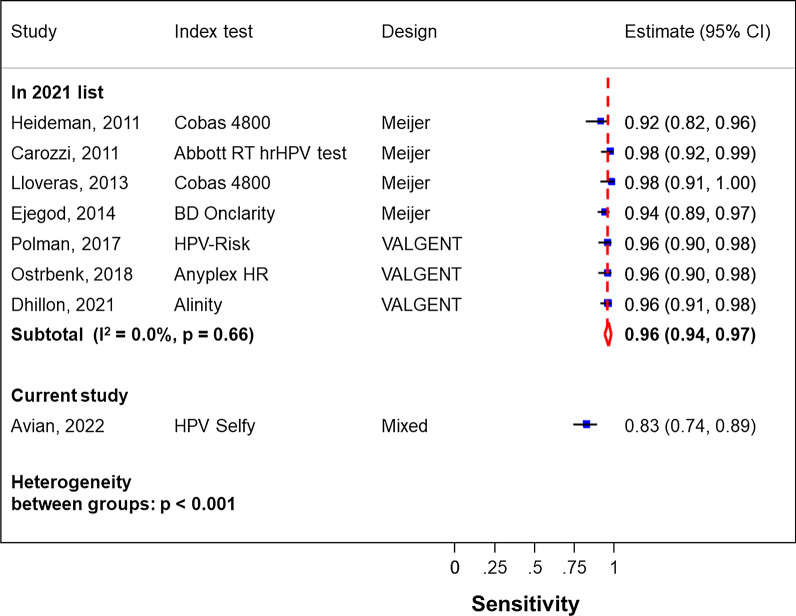
Sensitivity of the HC2 assay (standard comparator used in validation of hrHPV assays) in studies included in the 2020 list of HPV assays validated for cervical screening [5] that applied the Meier [2] or VALGENT [4] validation protocols (on top) or included in the study of Avian et al. [1] (at the bottom)

Validation requires an appropriately composed study population comprising a sufficient number of diseased subjects, derived from a continuous screening population or from a clearly described selection of CIN2+ cases and < CIN2 controls [4]. Avian et al. compiled cervical specimens for testing with HC2 (standard comparator test) and with the new *HPV Selfy* (index test) [1], but it remains unclear how the study population was composed. With 98 CIN2+ and 791 ≤ CIN1 subjects it was obviously not a continuous screening population, so more granularity on this would have been welcome. Additionally, detail on how non-disease was defined, which is essential for the evaluation of clinical specificity, was lacking. The reported absolute sensitivity for CIN2+ of the HC2 comparator test was 82.7%, which was substantially lower than the sensitivities observed in validation studies following the VALGENT or Meijer protocols included in a meta-analysis (Fig. 1) [5]. This may rise suspicion of a certain degree of histological over-classification. Nonetheless, we verified the data matrices in Table 2 in Avian et al. [1] and confirm the correctness of the non-inferiority statistics (Table 1).

**Table 1:** Computation of the relative specificity to exclude cervical intra-epithelial neoplasia of grade 2 or worse of Selfy on self-samples (SS) vs clinician-taken samples (clin) and non-inferiority statistics

Correct statistic			
	Selfy clin−	Selfy clin+	
Selfy SS−	708	16	724
Selfy SS+	37	30	67
	745	46	791
Specificity Selfy SS	= 724/791 =	91.5%	
Specificity Selfy clin	= 745/791 =	94.2%	
Relative specificity SS/clin	0.97	(95% CI 0.95–0.99)*	
T non inferiority	− 0.86		
p non-inferiority	0.81		

Wrong statistic (b and c cells switched in the abcd matrix)			
	Selfy clin−	Selfy clin+	
Selfy SS−	708	*37*	745
Selfy SS+	*16*	30	46
	724	67	791
Specificity Selfy clin	= 745/791 =	94.2%	
Specificity Selfy SS	= 724/791 =	91.5%	
Relative specificity clin/SS	1.03	(95% CI 1.01–1.05)*	
T non inferiority	*6.60*		
p non-inferiority	*< 0.0001*		

In italics: non-inferiority statistic reported by Avian et al. [1] which was due to erroneous switching the values 37 and 16. In fact this reported statistic reflects that Selfy on clin samples is not inferior to SS samples

The claim that *HPV Selfy* on self-samples was non-inferior to clinician-collected samples was flawed by critical statistical errors. The number of subjects with discordant self+ /clinician− and self−/clinician+ results (b and c cells in Table 4, in Avian et al. [1]) in the recommended formula for comparison of matched proportions were switched yielding reported p values < 0.05. Correct data entry would have generated non-inferiority p values 0.35 and 0.81 for sensitivity and specificity, respectively. The corresponding relative sensitivity and relative specificity for CIN2+ and 95% confidence intervals (not reported by authors) were 0.92 (95% CI 0.81–1.00) and 0.97 (95% CI 0.95–0.99), respectively, indicating non-significantly lower sensitivity and significantly lower specificity of *HPV Selfy* on self-versus clinician-collected samples.

Collaborations between science and industry are instrumental to advance clinical research, however contractual independency of researchers and autonomy of publication enhance scientific credibility. We observe that sixteen of thirty six authors (including the first and last) of the JTM paper are affiliated with the manufacturer of the assay. In the 2020 list of validated HPV assays [5], assays evaluated by test developers were down-graded to “partially validated” if all other validation criteria were fulfilled. This principle may also apply on the *HPV Selfy* assessment [1]. We recommend test developers, HPV experts and collaborating epidemiologists or statisticians to design validation studies according to internationally established protocols and evaluation methodologies. Journal editors should take this advice into account as well.
